# Development and validation of machine learning-based models for prediction of adolescent idiopathic scoliosis: A retrospective study

**DOI:** 10.1097/MD.0000000000033441

**Published:** 2022-04-07

**Authors:** Zheng Lv, Wen Lv, Lei Wang, Jiayuan Ou

**Affiliations:** a Department of Rehabilitation, Longgang District Central Hospital of Shenzhen, Shenzhen Clinical Medical College of Guangzhou University of Chinese Medicine, Shenzhen, Guangdong, China; b Department of Rehabilitation, Shenzhen People’s Hospital, The Second Clinical Medical College, Jinan University; The First Affiliated Hospital, Southern University of Science and Technology, Shenzhen, Guangdong, China.

**Keywords:** adolescent idiopathic scoliosis, machine learning, prediction model, risk

## Abstract

Adolescent idiopathic scoliosis (AIS) can cause abnormal body posture, which has a negative impact on the overall posture. Therefore, timely prevention and early treatment are extremely important. The purpose of this study is to build an early warning model of AIS risk, so as to provide guidance for accurately identifying early high-risk AIS children and adolescents. We conducted a retrospective study of 1732 children and adolescents with or without AIS who underwent physical examination in Longgang District Central Hospital of Shenzhen (LDCHS queue) from January 2019 to October 2022 and 1581 children and adolescents with or without AIS in Shenzhen People Hospital (January 2018 to December 2022) as external validation queues (SPH queue). The random forest model (RFM), support vector machine model, artificial neural network model (ANNM), decision tree model (DTM), and generalized linear model (GLM) were used to build AIS model for children and adolescents. The predictive efficacy of 5 machine learning models was evaluated by receiver operating characteristic curve and decision curve analysis. For screening candidate predictors of AIS, the ratio of sitting height to standing height (ROSHTSH), angle of lumbar rotation, scapular tilt (ST), shoulder-height difference (SHD), lumbar concave (LC), pelvic tilt (PT) and angle of thoracolumbar rotation (AOTR) can be used as a potential predictor of AIS. The effectiveness of the prediction model constructed by the 5 machine learning algorithms was between (area under the curve [AUC]: 0.767, 95% confidence interval [CI]: 0.710–0.824) and (AUC: 0.899, 95% CI: 0.842–0.956) in the training set and internal verification set, respectively. Among them, the ANNM was equipped with the best prediction effectiveness (training set: AUC: 0.899, 95% CI: 0.842–0.956) and (internal verification set: AUC: 0.897, 95% CI: 0.842–0.952). The prediction model of AIS based on machine learning algorithm can achieve satisfactory prediction efficiency, among which ANNM is the best, which can be used to guide clinicians in diagnosis and treatment and improve the prognosis of AIS children and adolescents.

## 1. Introduction

Adolescent idiopathic scoliosis (AIS, also known as scoliosis) is a kind of spinal deformity, including the changes of upper scoliosis on the coronal plane, thoracic cone kyphosis on the sagittal plane, and the rotation and wedge-shaped changes of the vertebral body on the horizontal plane.^[1,2]^ Worldwide, as a type with the highest probability of scoliosis, the incidence rate of AIS in adolescents is about 1% to 3%.^[3,4]^ It will not only affect the physiological function of adolescent children and adolescents, but also restrict the growth of children and adolescents’ height, affect their overall aesthetics, and then seriously affect the psychology of children and adolescents.^[3,5]^ Therefore, effective diagnostic methods should be adopted to prevent and treat AIS.

Up to now, idiopathic scoliosis mainly includes the following evaluation methods, namely, visual inspection, Adam forward bend test (FBT) and scoliometer measurement.^[3,6]^ However, there are some defects in these methods, especially due to excessive referrals leading to unnecessary frequent radiation examinations, which may cause further harm to the body of teenagers. Among them, the diagnosis of AIS is mainly based on the size of Cobb angle, which has strong clinical application value in disease classification, treatment plan formulation and prognosis evaluation. Generally, Cobb angle ≥ 10° indicates scoliosis, and Cobb angle < 25° requires regular review; 25° to 45° requires supportive treatment, while Cobb angle > 45° requires surgical correction.^[3,6,7]^ In view of this, screening the spinal growth curve of children and adolescents and adolescents, timely assessing the risk of scoliosis, and early warning are of great guiding value for the prevention and treatment of AIS.

Nowadays, thanks to the rapid development of artificial intelligence (AI) technology, the prediction model built by its branch machine learning algorithm has been proved to be widely used in the medical field, such as intelligent image recognition, intelligent health analysis, intelligent diagnosis, etc, promoting the unification of medical technology and improving the medical level.^[8,9]^ However, few studies have explored its predictive role in AIS. Compared with the traditional binary logistic regression model, the prediction model based on machine learning algorithm has higher diagnostic accuracy, which benefits from its high classification accuracy, strong parallel distributed processing ability, strong distributed storage and learning ability.^[10,11]^ In view of this, based on machine learning algorithm and the application of relevant indicators of scoliosis screening, this study intended to establish a risk prediction model for AIS based on machine learning to promote the implementation of early conservative treatment.

## 2. Materials and methods

### 2.1. Study participants

**Figure 1.**
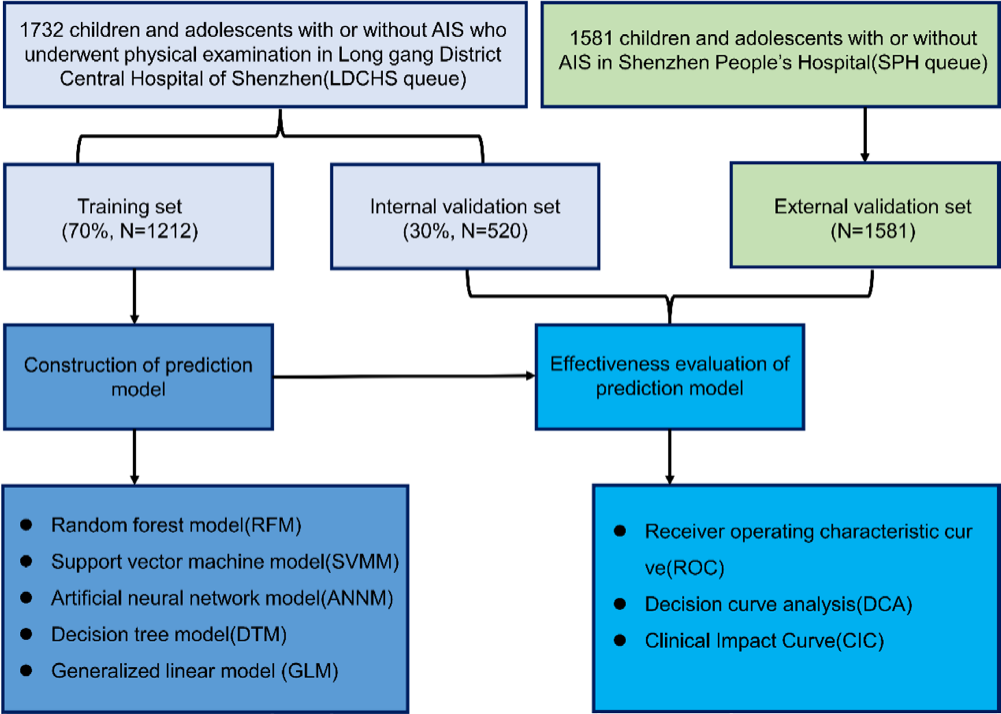
The process of participant inclusion and AIS prediction model construction. AIS = adolescent idiopathic scoliosis.

We conducted a retrospective study of 1732 children and adolescents with or without AIS who underwent physical examination in Longgang District Central Hospital of Shenzhen from January 2019 to October 2022 and 1581 children and adolescents with or without AIS in Shenzhen People Hospital (January 2018 to December 2022) as external validation queues. Inclusion criteria were as follows: Children and adolescents who volunteered for scoliosis screening; The parents or legal guardians of each student under the age of 18 years old need written or oral informed consent. Exclusion criteria: Children and adolescents unwilling to participate in screening and cooperate with physical examination (e.g., some people unwilling to let professional inspectors see their private parts of the body); Children and adolescents with protrusion and degeneration of intervertebral disc and other spinal or spinal diseases; Children and adolescents with clinical diagnosis of congenital scoliosis or neuromuscular scoliosis; Participants without informed consent. This retrospective study was approved by the Ethics Committee of Longgang District Central Hospital of Shenzhen, and the research scheme was implemented according to the AI model training specifications of the unit (EJCP20221021). All participants’ personal information is encrypted to prevent leakage and complies with the Declaration of Helsinki. All participants knew the content of the study and signed the informed consent form. The process of participant inclusion and prediction model construction was summarized in Figure 1.

### 2.2. Diagnostic criteria for AIS

**Figure 2.**
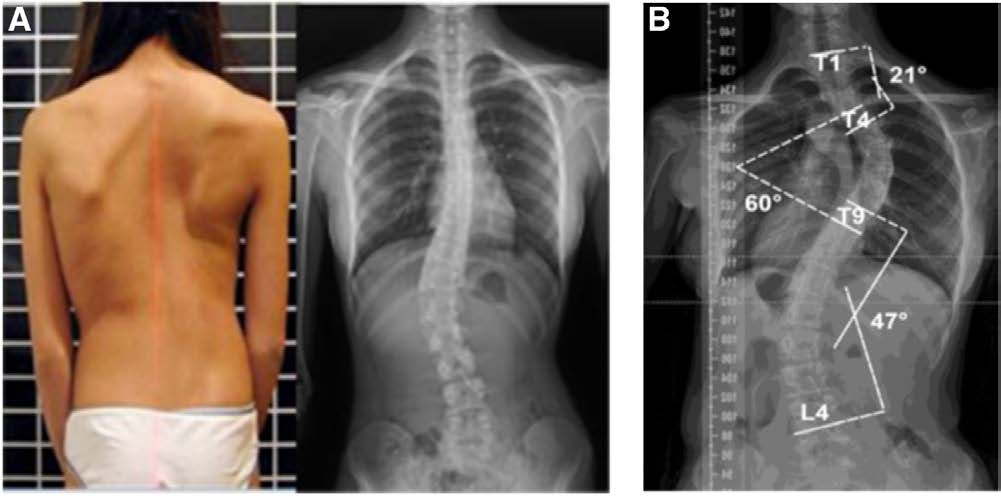
Diagnostic methods for children and adolescents with AIS. (A) All participants were screened for scoliosis in a closed room or tent, and the patients were placed in a standing position and received physical examination and evaluation by professionals; (B) When participants’ ATR is >5 ° or show obvious clinical signs of scoliosis, lateral Cobb angle examination is required to accurately measure spinal curvature. AIS = adolescent idiopathic scoliosis, ATR = angle of trunk rotation.

The scoliosis screening involved in this study was conducted by a team of experienced and trained rehabilitation therapists from Longgang District Central Hospital of Shenzhen, who used visual inspection, Adam FBT and measurement of angle of trunk rotation with a scoliometer for physical examination.^[12–14]^ When participants have trunk rotation angle > 5° or show obvious clinical signs of scoliosis, the lateral Cobb angle will be used to accurately measure the curvature of the spine. Cobb angle ≥ 10° was considered as AIS (as shown in Fig. 2), otherwise, it is considered as normal spine development appearance.^[15]^

### 2.3. Data entry and quality control of AIS

This study included the demographic characteristics of all participants, including gender, age (years), height and body mass index. The postural evaluation of participants mainly comes from visual inspection, Adam FBT and angle of trunk rotation to obtain correlation coefficients, namely: Ratio of sitting height to standing height (ROSHTSH), angle of lump rate (AOLR), scapular tilt (ST), shoulder height difference (SHD), lump construct (LC), pelvic tile (PT), angle of thoracolumbar rotation (AOTR), thoracic kyphosis, flat back (FB), and lumbar kyphosis.^[12]^ Among them, for the evaluation and measurement of the above indicators, in order to ensure the quality and reliability of the data obtained, all subjects were independently evaluated by 2 experts, and then the data were verified by a third expert. If there was data bias or error, the 3 experts jointly supervised the repeated testing, and finally the data measured by the 3 experts were included in the final study.

### 2.4. Construction and validation of prediction model based on logistic regression algorithm

According to whether the participant was finally diagnosed with AIS, we set AIS as “outcome variable,” and used the generalized linear model (GLM) algorithm, that is, establish the relationship between the mathematical expectation of the response variable and the prediction variable of the linear combination through the linkage function.^[16]^ At the same time, in order to ensure the best combination of variables included in GLM, we adopted the variable screening mode based on Akaike Information Criterion (AIC), that is, AIC can compare the relative difference/distance between each alternative model and the “real model,” the smaller the AIC value, the shorter the distance between the alternative model and the “real model” (the smaller the information loss).^[17]^

### 2.5. Construction and validation of prediction model based on machine learning algorithm

Based on the principles of different machine learning algorithms and public recognition, 4 common prediction models of machine learning algorithms are included in this study, namely, random forest model (RFM), support vector machine model, artificial neural network model (ANNM) and decision tree model (DTM). In short, RFM and DTM are based on the principle of “branching” to discriminate and classify each included variable; ANNM is based on the algorithms of “input layer,” “hidden layer” and “output layer” to build the optimal prediction efficiency of AIS; support vector machine model is a class of generalized linear classifiers that classify data in a binary way according to supervised learning. Its decision boundary is the maximum margin hyperplane for learning samples, which can turn the problem into a convex quadratic programming problem.

### 2.6. Effectiveness evaluation of predictive model for AIS

In this study, the predictive efficacy of 4 machine learning models and GLM were evaluated by receiver operating characteristic curve and decision curve analysis. In addition, we also used the continuous correction curve to evaluate the robustness of GLM, and the clinical impact curve to evaluate the differentiation efficiency of the optimal prediction model (i.e., ANNM). All prediction models were tested in training set, internal verification set and external verification set.

### 2.7. Statistical analysis

The data analysis and visualization involved in this study were completed using R software (download address: https://www.r-project.org/). For descriptive analysis, the median (inter-quartile range) and frequency (%) of continuous variables and categorical variables were evaluated respectively. Pearson correlation coefficient was used to measure the degree of correlation between each variable, and the Least Absolute Shrinkage and Selection Operator regression was used for model variable screening and complexity adjustment. In the process of variable screening and inter group comparison, *P* values <.05 were considered as statistically significant.

## 3. Results

### 3.1. Baseline characteristics of AIS cohort and non-AIS cohort

Participants were divided into AIS group and non-AIS group according to whether they were finally diagnosed with AIS. In order to build the AIS prediction model, we adopted a random division method (i.e., 70% of the training sets and 30% of the internal validation sets), in which the AIS proportion in the training set is 12.29%, while the AIS proportion in the internal validation set is 13.08% (from LDCHS queue). In addition, 1581 participants from Shenzhen People Hospital (SPH queue) were considered as external validation queues, with AIS accounting for 11.57%. From the comparison between the AIS group and the non-AIS group in the internal queue, there were significant differences between groups (*P* < .05) in the ROSHTSH, AOLR, ST, SHD, LC, PT, AOTR, thoracic kyphosis, FB, and lumbar kyphosis (*P* < .05), the same trend appears in external queues. The baseline data of all participants were summarized in Table 1 and Table S1, Supplemental Digital Content 1, http://links.lww.com/MD/I747.

**Table 1:** Demographic characteristics of participants and comparison by AIS stratification (from LDCHS queue).

Variables	Training cohort			*P* value	Internal validation cohort			*P* value
	Overall (N = 1212)	AIS (N = 149)	Non-AIS (N = 1063)		Overall (N = 520)	AIS (N = 68)	Non-AIS (N = 452)	
Gender (%)								
Male	616 (50.8)	65 (43.6)	551 (51.8)	.073	265 (51.0)	34 (50.0)	231 (51.1)	.968
Female	596 (49.2)	84 (56.4)	512 (48.2)		255 (49.0)	34 (50.0)	221 (48.9)	
Age (median [IQR])	13.00 [11.00, 15.00]	12.00 [11.00, 14.00]	13.00 [11.00, 15.00]	.015	13.00 [11.00, 15.00]	12.00 [11.00, 14.00]	13.00 [11.00, 15.00]	.01
BMI (median [IQR]), kg/m^2^	22.60 [20.40, 24.90]	22.20 [20.60, 24.20]	22.60 [20.40, 24.90]	.271	22.80 [20.50, 24.90]	22.80 [20.88, 24.70]	22.75 [20.38, 24.90]	.877
ROSHTSH (median [IQR])	0.58 [0.56, 0.61]	0.52 [0.48, 0.54]	0.59 [0.57, 0.61]	<.001	0.59 [0.56, 0.61]	0.51 [0.49, 0.54]	0.59 [0.57, 0.61]	<.001
AOLR (%)								
Normal	905 (74.7)	11 (7.4)	894 (84.1)	<.001	378 (72.7)	6 (8.8)	372 (82.3)	<.001
Rotate to the left	185 (15.3)	80 (53.7)	105 (9.9)		98 (18.8)	48 (70.6)	50 (11.1)	
Rotate to the right	122 (10.1)	58 (38.9)	64 (6.0)		44 (8.5)	14 (20.6)	30 (6.6)	
ST (%)								
Normal	1016 (83.8)	15 (10.1)	1001 (94.2)	<.001	423 (81.3)	8 (11.8)	415 (91.8)	<.001
Tilt to the left	122 (10.1)	82 (55.0)	40 (3.8)		48 (9.2)	34 (50.0)	14 (3.1)	
Tilt to the right	74 (6.1)	52 (34.9)	22 (2.1)		49 (9.4)	26 (38.2)	23 (5.1)	
SHD (%)								
Normal	906 (74.8)	11 (7.4)	895 (84.2)	<.001	393 (75.6)	4 (5.9)	389 (86.1)	<.001
Left shoulder height	167 (13.8)	87 (58.4)	80 (7.5)		71 (13.7)	44 (64.7)	27 (6.0)	
Right shoulder height	139 (11.5)	51 (34.2)	88 (8.3)		56 (10.8)	20 (29.4)	36 (8.0)	
LC (%)								
Normal	962 (79.4)	14 (9.4)	948 (89.2)	<.001	401 (77.1)	5 (7.4)	396 (87.6)	<.001
Left concave	89 (7.3)	54 (36.2)	35 (3.3)		54 (10.4)	34 (50.0)	20 (4.4)	
Right concave	161 (13.3)	81 (54.4)	80 (7.5)		65 (12.5)	29 (42.6)	36 (8.0)	
PT (%)								
Normal	993 (81.9)	6 (4.0)	987 (92.9)	<.001	423 (81.3)	5 (7.4)	418 (92.5)	<.001
Tilt to the left	125 (10.3)	78 (52.3)	47 (4.4)		56 (10.8)	40 (58.8)	16 (3.5)	
Tilt to the right	94 (7.8)	65 (43.6)	29 (2.7)		41 (7.9)	23 (33.8)	18 (4.0)	
AOTR (%)								
Normal	1083 (89.4)	101 (67.8)	982 (92.4)	<.001	446 (85.8)	49 (72.1)	397 (87.8)	.001
Rotate to the left	62 (5.1)	27 (18.1)	35 (3.3)		34 (6.5)	11 (16.2)	23 (5.1)	
Rotate to the right	67 (5.5)	21 (14.1)	46 (4.3)		40 (7.7)	8 (11.8)	32 (7.1)	
TK (%)								
Normal	1059 (87.4)	28 (18.8)	1031 (97.0)	<.001	445 (85.6)	8 (11.8)	437 (96.7)	<.001
Abnormal	153 (12.6)	121 (81.2)	32 (3.0)		75 (14.4)	60 (88.2)	15 (3.3)	
FB (%)								
Normal	1145 (94.5)	95 (63.8)	1050 (98.8)	<.001	493 (94.8)	45 (66.2)	448 (99.1)	<.001
Abnormal	67 (5.5)	54 (36.2)	13 (1.2)		27 (5.2)	23 (33.8)	4 (0.9)	
LK (%)								
Normal	1180 (97.4)	125 (83.9)	1055 (99.2)	<.001	509 (97.9)	61 (89.7)	448 (99.1)	<.001
Abnormal	32 (2.6)	24 (16.1)	8 (0.8)		11 (2.1)	7 (10.3)	4 (0.9)	

AIS = adolescent idiopathic scoliosis, AOLR = angle of lumbar ratation, AOTR = angle of thoracolumbar rotation, BMI = Body mass index, FB = flat back, IQR = inter-quartile range, LC = lumbar concave, LDCHS = Longgang District Central Hospital of Shenzhen, LK = lumbar kyphosis, PT = pelvic tilt, ROSHTSH = ratio of sitting height to standing height, SHD = shoulder-height difference, ST = scapular tilt, TK = thoracic kyphosis.

### 3.2. Selection of candidate predictive features for AIS

**Figure 3.**
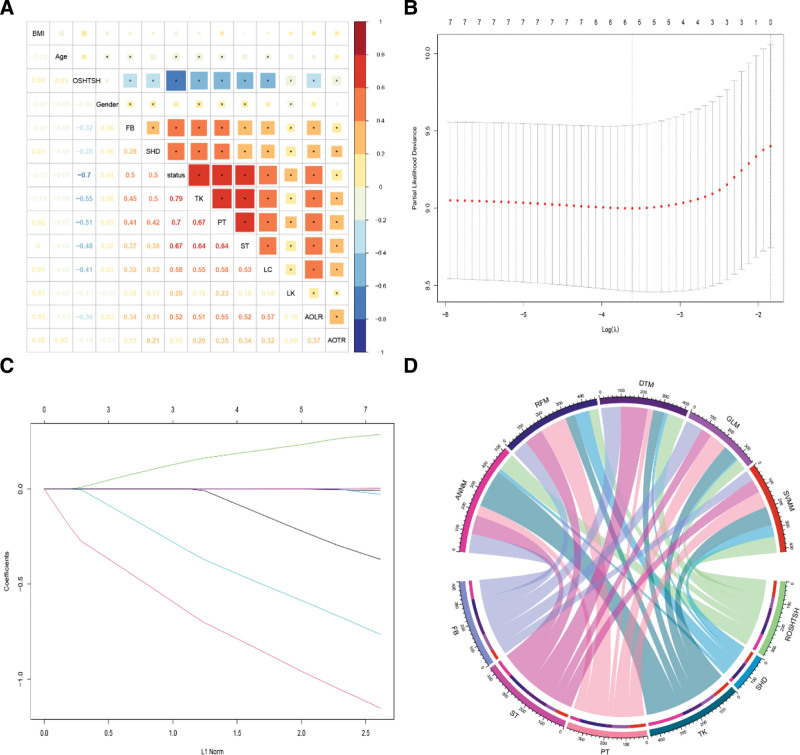
Selection of candidate variables for AIS prediction model. (A) Screening AIS candidate variables based on Pearson correlation coefficient; (B) Lasso variable trajectory spectrum for features with missing rate <5% in the candidate queue; (C) Feature selection by LASSO; (D) Weight distribution of candidate variables (top 6) in various prediction models. AIS = adolescent idiopathic scoliosis, LASSO = least absolute shrinkage and selection operator.

A total of 13 candidate variables were included in the machine learning algorithm screening process. First, we used correlation analysis, that is, the correlation matrix composed of AIS and 13 candidate variables to show the correlation strength of each variable, as shown in Figure 3. Secondly, we further used least absolute shrinkage and selection operator regression to recursively calculate the candidate variables, and found that only 6 candidate variables showed significant positive or negative correlation with AIS, namely, ROSHTSH, ST, SHD, PT, FB, and TK. To sum up, relevant candidate factors based on scoliosis screening may play a certain role in predicting AIS.

### 3.3. Construction of predictive model for AIS based on machine learning algorithm

**Figure 4.**
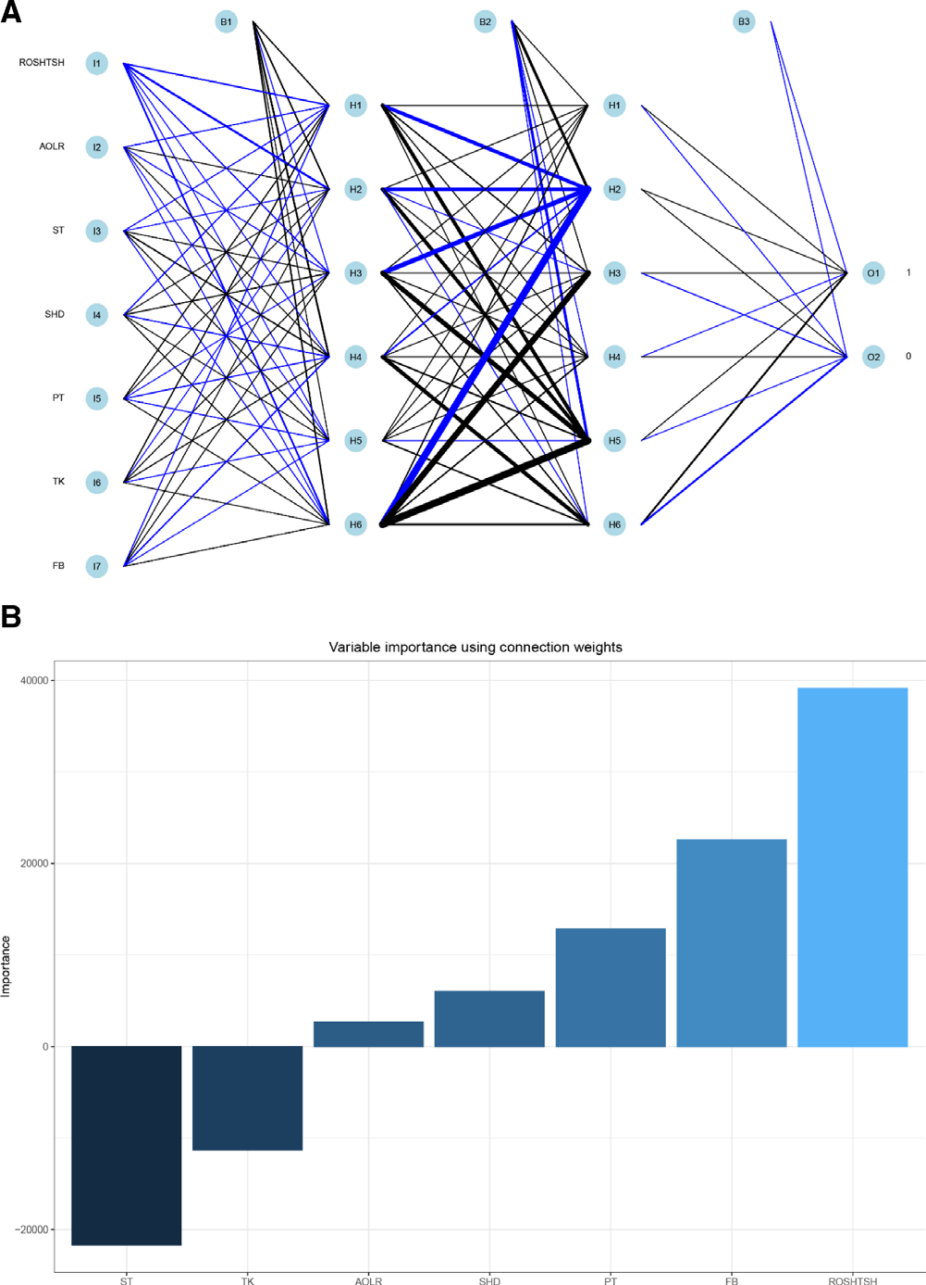
AIS prediction model based on ANN algorithm. (A) Visualization of ANNM and subsequent variable calculus; (B) Variable importance using connection weights. AIS = adolescent idiopathic scoliosis, ANN = artificial neural network, ANNM = artificial neural network model.

In order to further evaluate the performance of candidate variables in 5 models, we built 4 types of models based on machine learning algorithms. First, we used the ANNM algorithm to include 8 potential prediction factors, as shown in Figure 4A. After the candidate variables pass through the “input layer,” they can complete at least 2 “hidden layer” iterations, and then pass through the “output layer” to obtain the AIS interpretation results. The weight distribution of each prediction factor was different, as shown in Figure 4B. Secondly, we built RFM and DTM based on the “bagging” algorithm, as shown in Table S2, Supplemental Digital Content 2, http://links.lww.com/MD/I748 and Figure S1, Supplemental Digital Content 3, http://links.lww.com/MD/I749. ROSHTSH, ST, SHD, PT, FB, and TK also play an important role in the prediction of RFM and DTM, especially ROSHTSH and TK can be the intersection predictor of RFM and DTM, which further reflected the similarity of the 2 algorithms. Finally, we also calculated SVM and GLM. As far as compatibility was concerned, after GLM was included in the above candidate variables, the weight of each prediction variable is inconsistent, as shown in Figure S2, Supplemental Digital Content 4, http://links.lww.com/MD/I750, and even if resampling analysis was carried out, it clearly showed that GLM algorithm has a certain degree of robustness. This showed that it is feasible to build AIS prediction models based on scoliosis screening, and the robustness of different models must be further evaluated in the internal verification set and external queues.

### 3.4. Effectiveness evaluation of machine learning-based prediction models

**Figure 5.**
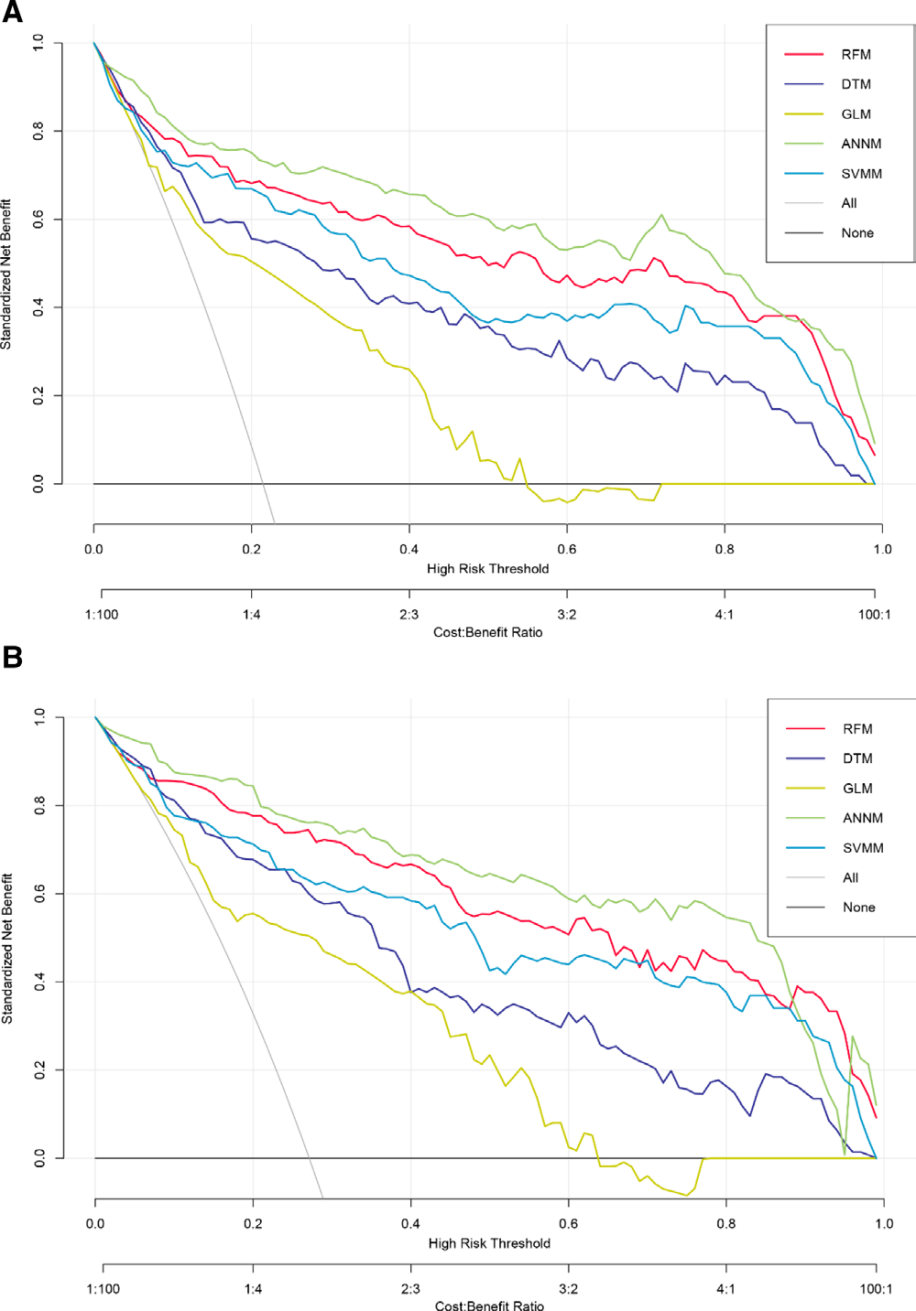
Evaluation of prediction effectiveness of 5 AIS prediction models. (A) Training cohort; (B) Internal validation cohort. AIS = adolescent idiopathic scoliosis.

Next, to further evaluate the robustness and accuracy of 5 prediction models in predicting AIS, we used decision curve analysis to evaluate the effectiveness of each prediction model. As shown in Figure 5, the robustness of ANNM was significantly better than the other 4 prediction models in both training set and validation set queues, which shows that ANNM has certain advantages in predicting AIS. At the same time, we also used the receiver operating characteristic (i.e., area under the curve [AUC]) to evaluate the accuracy. The results showed that the predictive performance of ANNM in training set and internal verification set was (AUC: 0.899, 95% confidence interval [CI]: 0.842–0.956) and (AUC: 0.897, 95% CI: 0.842–0.952), respectively, followed by the RFM model (training set: [AUC: 0.863, 95% CI: 0.806–0.920]; verification set: [AUC: 0.875, 95% CI: 0.820–0.930]), as shown in Table 2. In general, the prediction model based on 5 different algorithms has good prediction efficiency and can provide risk stratification guidance for children and adolescents with or without AIS.

**Table 2:** Evaluating the effectiveness of 5 AIS prediction models based on the AUC of ROC.

Model	Training cohort				Internal validation cohort				External validation cohort			
	AUC	95% CI	PPV	NPV	AUC	95% CI	PPV	NPV	AUC	95% CI	PPV	NPV
ANNM	0.899	0.842–0.956	97.32%	99.53%	0.897	0.842–0.952	95.59%	99.12%	0.889	0.834–0.944	98.36%	99.71%
RFM	0.863	0.806–0.920	95.97%	97.84%	0.875	0.820–0.930	92.65%	97.57%	0.872	0.817–0.927	95.62%	98.14%
SVMM	0.827	0.770–0.884	87.92%	96.33%	0.836	0.781–0.891	88.24%	96.90%	0.823	0.768–0.878	90.16%	97.21%
DTM	0.815	0.758–0.872	85.23%	95.77%	0.824	0.769–0.879	85.29%	95.35%	0.819	0.764–0.874	87.43%	96.92%
GLM	0.767	0.710–0.824	82.55%	95.30%	0.771	0.716–0.826	83.82%	94.91%	0.768	0.713–0.824	84.70%	95.78%

95% CI = 95% confidence interval, ANNM = artificial neural network model, AUC = area under the curve, DTM = decision tree model, GLM = generalized linear model, NPV = negative predictive value, PPV = positive predictive value, RFM = random forest model, SVMM = support vector machine model.

### 3.5. External queue validation of AIS prediction models

**Figure 6.**
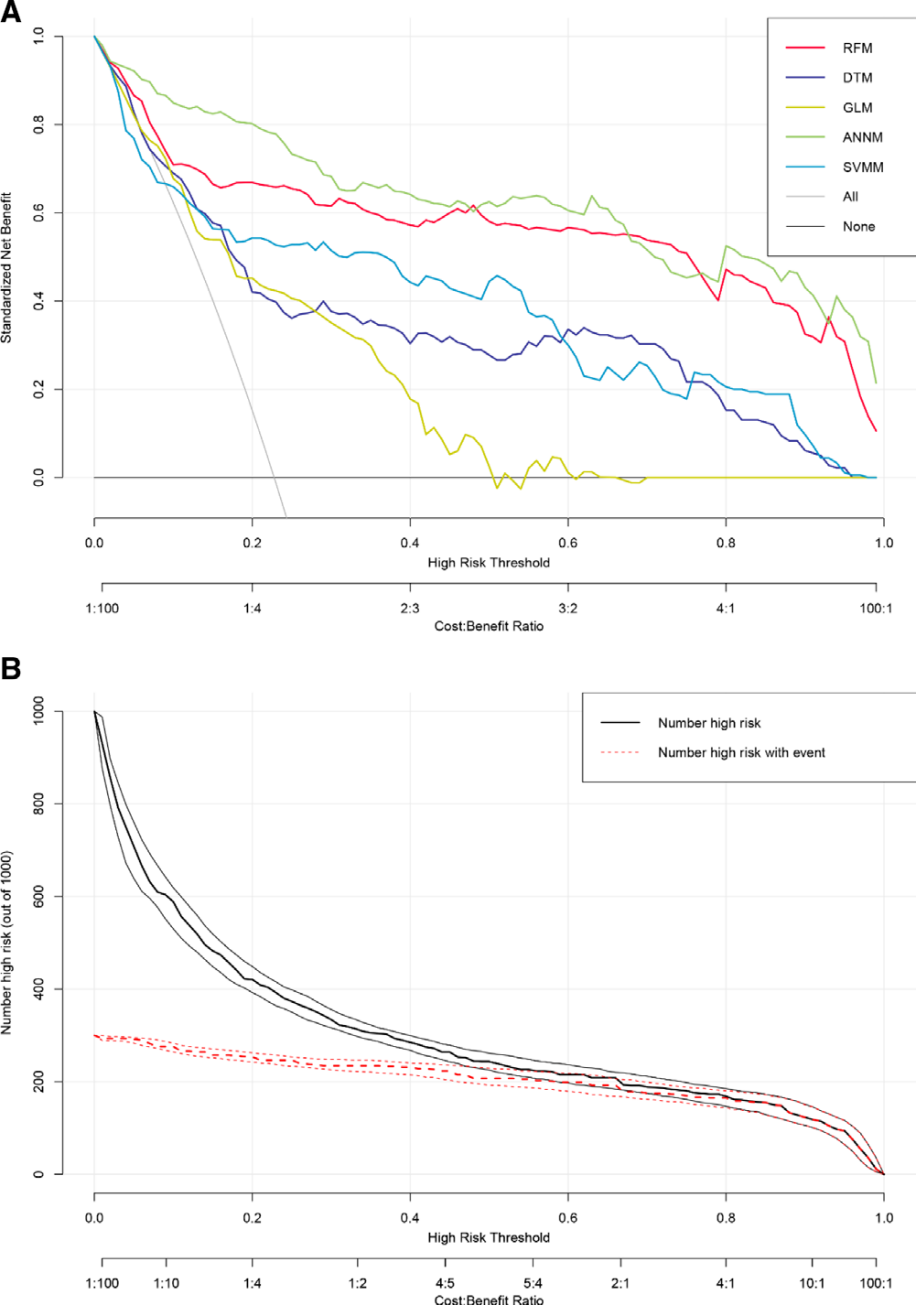
Validation of 5 AIS prediction models in external queue. (A) Prediction effectiveness evaluation of AIS prediction model; (B) AIS discrimination efficiency of ANNM. AIS = adolescent idiopathic scoliosis, ANNM = artificial neural network model.

The above research results show that ANNM has the best prediction efficiency among the models constructed by the 5 algorithms. In order to further test whether its external prediction has the same robustness and accuracy, we have included the data of Shenzhen People Hospital for external calculation verification. As shown in Figure 6A, the prediction models of 5 machine learning algorithms still have consistent robustness in external queues. Similarly, we also used the optimal prediction model (i.e., ANNM) to predict the hierarchical risk of AIS in the external cohort, as shown in Figure 6B. Even in the external cohort, ANNM can also identify whether participants have AIS risk, which confirmed that the prediction model of AIS had reliable scalability.

## 4. Discussion

Idiopathic scoliosis is a scoliosis caused by unknown reasons during the growth and development of children and adolescents.^[18]^ It has become the third “killer” facing children and adolescents after obesity and myopia. Previous studies have shown that 0.47% to 5.2% of children and adolescents aged 10 to 18 years have idiopathic scoliosis, and 80% to 90% of them are female.^[18,19]^ It is worth mentioning that 27% to 59% of AIS children and adolescents may be accompanied by weakness of limb muscle strength, loss of function and respiratory muscle function in addition to pain symptoms, so it is extremely urgent to identify and take active and effective measures to intervene as soon as possible.^[20,21]^ At present, although the impact mechanism of growth and development on AIS is not very clear, people generally agree that the growth and development mode of spine bones is related to the size of scoliosis radian.^[22]^ Therefore, the use of variables related to scoliosis radian to construct the risk of adolescent scoliosis can effectively help predict the size of scoliosis angle in children and adolescents with mild and moderate AIS, so as to carry out early intervention.

Compared with previous studies, this study can provide an early warning for AIS and establish effective prevention and control strategies. In addition, the prediction model built by the deep learning algorithm can break the traditional Cobb angle diagnosis of AIS in addition to the convenience of traditional measurement indicators, which shows that the prediction model is more accurate and therefore more objective, so it is more suitable for the diagnosis of AIS in clinical practice. As far as we know, this is the first time that we have used the machine learning prediction model with clinically relevant variables to improve its performance, which helps to early identify suspected AIS children and adolescents, and provides early warning for timely intervention and treatment of these high-risk groups.

Until now, researchers have been trying to find predictors that can be used to predict AIS. Previous systematic reviews have shown that AIS can be identified through Adam FBT, scoliometer, or a combination of both, as well as the data of clinical signs and torso angle visual examination, but the low prediction efficiency caused by its false positive rate cannot be ignored.^[23,24]^ At the same time, some researchers studied the measurement of Cobb angle based on depth neural network and pointed out that the region of interest where the upper and lower cones are located can be manually selected, and then the corresponding slope of the spine can be automatically estimated to complete the automatic solution of Cobb angle, but manual operation will increase the learning cost. In addition, many studies are devoted to improving the accuracy and consistency of Cobb angle measurement.^[25,26]^ It is undeniable that the severity, progress evaluation and surgical indications of scoliosis require measurement of Cobb angle of scoliosis.^[27]^ In contrast, it seems that it is better to intervene in advance to discuss whether the signs of incorrect postures commonly used by children and adolescents can effectively predict the occurrence of AIS, rather than to evaluate after the skeletal development of children and adolescents is mature, then take invasive surgical treatment and the related surgical risks and postoperative complications that may be undertaken.

Fortunately, a few studies have made efforts to explore the early warning of AIS risk. For example, Yan et al built a prediction model for AIS based on binary linear regression algorithm, and the prediction accuracy can reach 82.57% to 83.30%^[12]^; Xu et al developed a genetic prediction model that can be used to assess the discrimination between AIS children and adolescents and normal control groups.^[28]^ Nault et al established a prediction model using a general linear method, taking 3D spinal parameters and clinical parameters as predictors, and found that the positive predictive value of the model was 79% (provided that the Cobb angle was 35° as the critical point).^[29]^ The idea of this study is basically consistent with that of previous studies, both of which are committed to improving the prediction efficiency of AIS risk. We analyzed and established the prevalence of incorrect posture in children and adolescents with AIS and established a prediction model based on machine learning algorithms with different adjustment weights to improve the prediction accuracy and provide targeted prevention strategies for AIS.

In this study, the candidate variables screened by various machine learning algorithms showed that ROSHTSH, AOLR, ST, SHD, LC, PT, and AOTR were the most valuable candidate variables for AIS prediction model, which was consistent with previous research results.^[12]^ Interestingly, previous studies have shown that the height of children and adolescents with scoliosis increases rapidly, with the average height growth rate of female AIS children and adolescents peaking at 8.1 cm/year, while the average height growth rate of normal female children and adolescents of the same age peaking at 7.1 cm/year.^[1,30]^ Therefore, it is believed that the growth and development of AIS children and adolescents at the peak of growth and development is faster than that of normal children and adolescents of the same age. It can be inferred from this study that with the change of the ratio of ROSHTSH of AIS children and adolescents, their Cobb angle also changes. Although the children and adolescents with large lateral bending angle, their sitting height decreases greatly due to the influence of lateral bending radian. Therefore, for mild and moderate AIS children and adolescents, with the increase of the ROSHTSH of different children and adolescents, the lateral bending angle will also increase accordingly. As for AOLR, ST, SHD, LC, PT, and AOTR, some researchers speculate that changes in the scapula and lumbar spine may be considered as adaptive compensation or muscle activation strategies for AIS children and adolescents, which is consistent with the results of this study.^[12,31,32]^ We are encouraged that these follow-up variables are relatively easy to obtain and have no negative effects such as radiation, so we can often have more advantages in the process of building the AIS early warning model.

It is undeniable that the prediction model built by machine learning algorithm really has a more significant advantage in AIS risk assessment. The advantages of machine learning model can be summarized as follows: first, machine learning algorithm can identify data trends and patterns that may be missed by human; Second, the machine learning prediction model can operate without human intervention after setting; Thirdly, the prediction model built by machine learning will become more and more accurate over time; Finally, machine learning algorithms can process various data formats in dynamic, large-capacity and complex data environments. Compared with traditional linear regression models, machine learning algorithm often has more robust output results, which benefits from the improvement of the algorithm. Especially for the same candidate variables, after repeated iteration of machine learning, its prediction efficiency tends to be more robust, this is because variables in the “hidden layer” have been verified by multiple iterations, so better prediction efficiency can be obtained.^[33,34]^ Therefore, the ANNM of the 5 machine learning prediction models included in this study is the best, whether in the internal training set or in the external verification queue, followed by RFM, which also confirms that the practical value of machine learning algorithms in AIS is beyond doubt.

Although we strive to build an AIS prediction model, our study inevitably has some limitations. First, this study belongs to a single center retrospective study. Although there are data from affiliated hospitals as external validation, the prediction model still needs to be repeatedly validated by large sample data from multiple centers. Second, this study is based on visual sign evaluation and has not conducted research on genomics of children and adolescents. In the future, it is still necessary to explore new candidate variables from the perspective of multiple genomics and predict AIS in multiple dimensions. Third, this study has not yet conducted grading prediction on the severity of AIS. In the future, follow-up should be carried out for AIS children and adolescents in order to build an early warning and prognosis prediction model for AIS.

## 5. Conclusion

Altogether, based on the routine scoliosis screening and machine learning algorithm, we have developed a stable and powerful feature to assess AIS risk. This AIS model is a very promising tool, in which ANMM has a high diagnostic efficiency and can optimize the decision-making and monitoring protocol of AIS children and adolescents.

## Acknowledgments

The researchers thanked all the participants in this study and offered their health data free of charge.

## Author contributions

**Conceptualization:** Zheng Lv.

Formal analysis: Lei Wang.

Methodology: Zheng Lv.

Resources: Zheng Lv, Ou Jiayuan.

Software: Lei Wang, Ou Jiayuan.

Supervision: Ou Jiayuan.

Visualization: Wen Lv, Ou Jiayuan.

Writing – original draft: Wen Lv, Ou Jiayuan.

Writing – review & editing: Ou Jiayuan.

## Supplementary Material
