# Supplementary material for: Development and validation of machine learning-based models for prediction of adolescent idiopathic scoliosis: A retrospective study
Source: Medicine (Baltimore). 2022 Apr 7;102(14):e33441. doi: 10.1097/MD.0000000000033441 (PMC10082234; doi:10.1097/MD.0000000000033441)
Supplement: Supplementary file 1 [file medi-102-e33441-s001.pdf]

Supplementary Table1. Demographic characteristics of participants and comparison by AIS stratification(from SPH queue)

Variables	Overall(N=1581)	AIS(N=183)	Non-AIS(N=1398)	P-value
Gender (%)				
Male	285 (18.0)	30 (16.4)	255 (18.2)	0.611
Female	1296 (82.0)	153 (83.6)	1143 (81.8)	
Age (median [IQR])	13.00 [11.00, 15.00]	12.00 [11.00, 14.00]	13.00 [11.00, 15.00]	0.002
BMI (median [IQR]),kg/m2	22.60 [20.40, 24.90]	22.30 [20.65, 24.20]	22.60 [20.30, 24.90]	0.48
ROSHTSH (median [IQR])	0.58 [0.56, 0.61]	0.52 [0.48, 0.54]	0.59 [0.57, 0.61]	<0.001
AOLR (%)				
Normal	1184 (74.9)	23 (12.6)	1161 (83.0)	<0.001
Rotate to the left	239 (15.1)	88 (48.1)	151 (10.8)	
Rotate to the right	158 (10.0)	72 (39.3)	86 (6.2)	
ST (%)				
Normal	1330 (84.1)	25 (13.7)	1305 (93.3)	<0.001
Tilt to the left	141 (8.9)	89 (48.6)	52 (3.7)	
Tilt to the right	110 (7.0)	69 (37.7)	41 (2.9)	
SHD (%)				
Normal	1273 (80.5)	18 (9.8)	1255 (89.8)	<0.001
Left shoulder height	196 (12.4)	106 (57.9)	90 (6.4)	
Right shoulder height	112 (7.1)	59 (32.2)	53 (3.8)	
LC (%)				
Normal	1254 (79.3)	19 (10.4)	1235 (88.3)	<0.001
Left concave	128 (8.1)	78 (42.6)	50 (3.6)	
Right concave	199 (12.6)	86 (47.0)	113 (8.1)	
PT (%)				
Normal	1315 (83.2)	14 (7.7)	1301 (93.1)	<0.001
Tilt to the left	136 (8.6)	81 (44.3)	55 (3.9)	
Tilt to the right	130 (8.2)	88 (48.1)	42 (3.0)	
AOTR (%)				
Normal	1398 (88.4)	130 (71.0)	1268 (90.7)	<0.001
Rotate to the left	84 (5.3)	31 (16.9)	53 (3.8)	

Rotate to the right	99 (6.3)	22 (12.0)	77 (5.5)	
TK (%)				
Normal	1398 (88.4)	47 (25.7)	1351 (96.6)	<0.001
Abnormal	183 (11.6)	136 (74.3)	47 (3.4)	
FB (%)				
Normal	1480 (93.6)	97 (53.0)	1383 (98.9)	<0.001
Abnormal	101 (6.4)	86 (47.0)	15 (1.1)	
LK (%)				
Normal	1547 (97.8)	154 (84.2)	1393 (99.6)	<0.001
Abnormal	34 (2.2)	29 (15.8)	5 (0.4)	

Abbreviations: IQR. inter-quartile range;BMI. Body mass index; ROSHTSH.Ratio of sitting height to standing height;AOLR.angle of lumbar ratation;ST.scapular tilt;SHD.shoulder-height difference;LC.lumbar concave;PT.pelvic tilt;AOTR.angle of thoracolumbar rotation;TK.thoracic kyphosis;FB.flat back;LK.lumbar kyphosis.
