# Supplementary material for: Development and validation of machine learning-based models for prediction of adolescent idiopathic scoliosis: A retrospective study
Source: Medicine (Baltimore). 2022 Apr 7;102(14):e33441. doi: 10.1097/MD.0000000000033441 (PMC10082234; doi:10.1097/MD.0000000000033441)
Supplement: Supplementary file 2 [file medi-102-e33441-s002.pdf]

Supplementary Table2. Ranking of weight proportion of candidate variables based on RFM

Variables	%IncMSE	IncNodePurity
Gender	13.09967	1.785995
Age	17.604	2.079604
BMI	2.796135	2.023307
ROSHTSH	91.45182	77.27887
AOLR	17.04011	3.793277
ST	19.21505	17.30787
SHD	30.48054	11.45177
LC	16.48906	8.739632
PT	21.55161	21.69266
AOTR	4.628028	1.270792
TK	21.64806	33.51947
FB	17.54563	4.247771
LK	11.16983	1.387691

Abbreviations: ROSHTSH.Ratio of sitting height to standing height;AOLR.angle of lumbar ratation;ST.scapular tilt;SHD.shoulder-height difference;LC.lumbar concave;PT.pelvic tilt;AOTR.angle of thoracolumbar rotation;TK.thoracic kyphosis;FB.flat back;LK.lumbar kyphosis.
