# Supplementary material for: Development and validation of machine learning-based models for prediction of adolescent idiopathic scoliosis: A retrospective study
Source: Medicine (Baltimore). 2022 Apr 7;102(14):e33441. doi: 10.1097/MD.0000000000033441 (PMC10082234; doi:10.1097/MD.0000000000033441)
Supplement: Supplementary file 3 [file medi-102-e33441-s003.pdf]

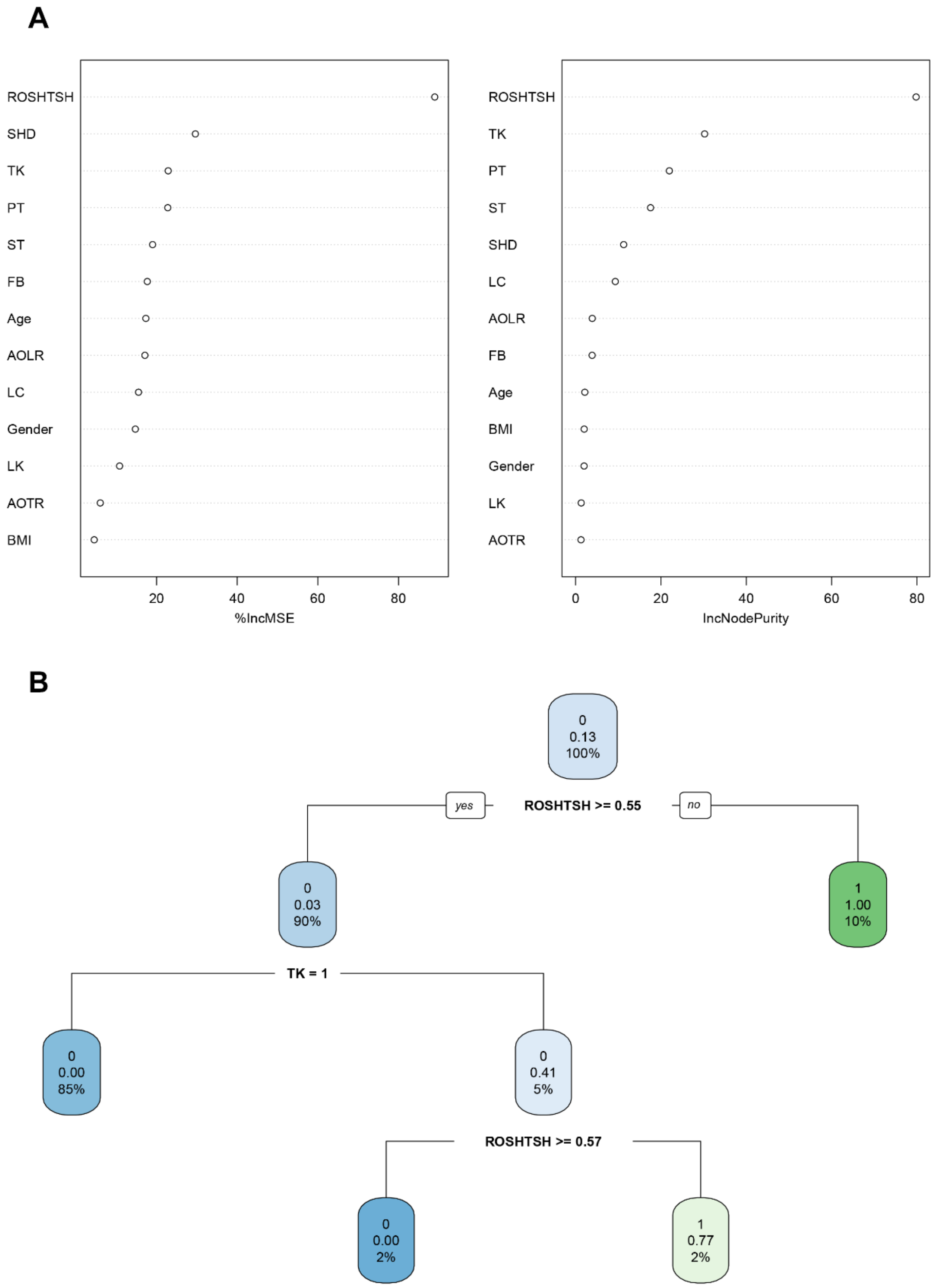

Supplementary Figure1. Visualization of AIS prediction model based on "bagging" algorithm.A.RFM;B.DTM.
