# Supplementary material for: Development and validation of machine learning-based models for prediction of adolescent idiopathic scoliosis: A retrospective study
Source: Medicine (Baltimore). 2022 Apr 7;102(14):e33441. doi: 10.1097/MD.0000000000033441 (PMC10082234; doi:10.1097/MD.0000000000033441)
Supplement: Supplementary file 4 [file medi-102-e33441-s004.pdf]

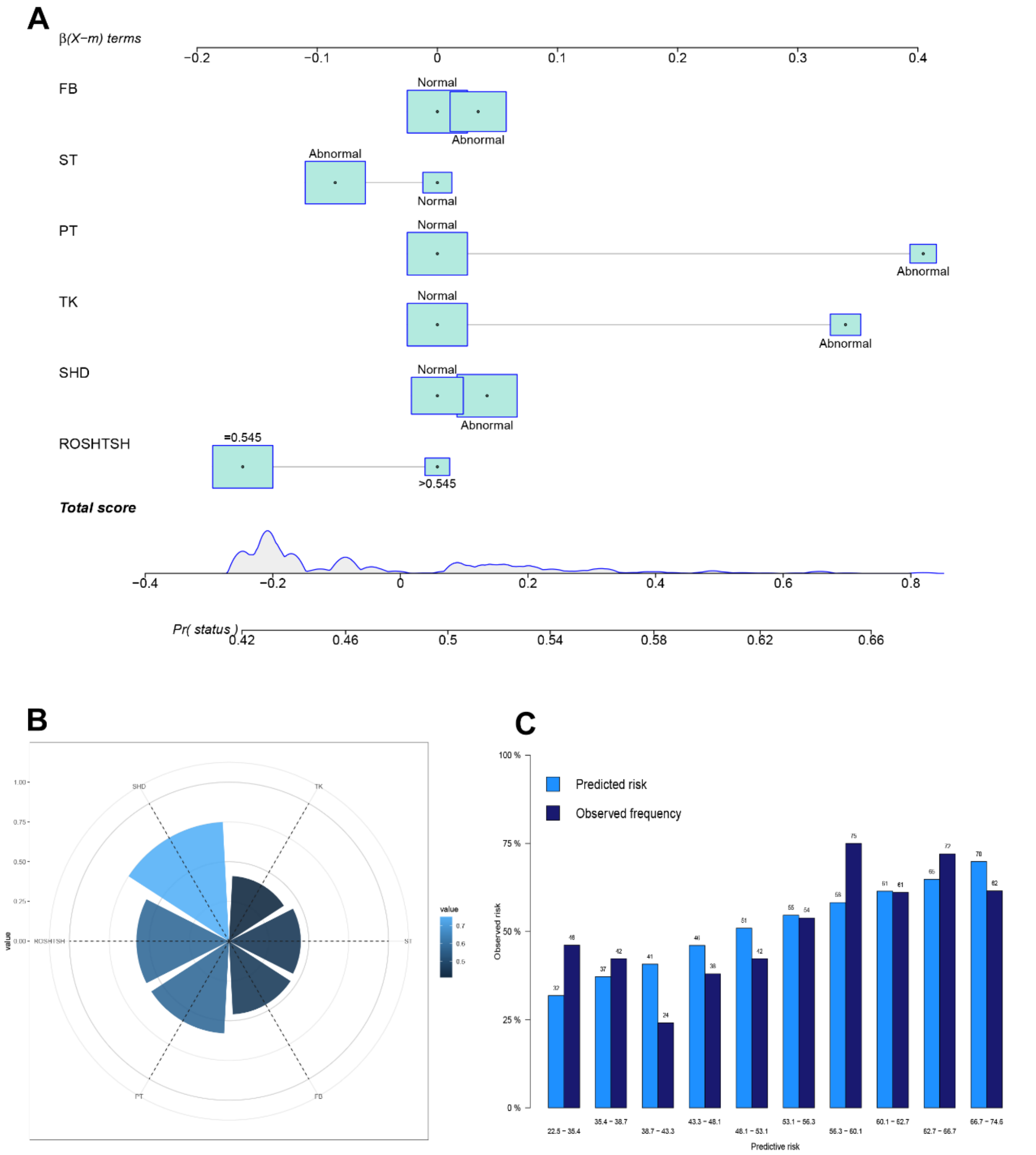

Supplementary Figure2. AIS prediction model based on generalized linear regression algorithm.A.Visualization of GLM;B.Proportion of predicted variable weight input in GLM;C.Robustness effectiveness evaluation of GLM in internal training set (based on repeated sampling).
